# Feasibility and acceptability of an acceptance and commitment therapy intervention for caregivers of adults with Alzheimer’s disease and related dementias

**DOI:** 10.1186/s12877-021-02078-0

**Published:** 2021-02-16

**Authors:** Nicole R. Fowler, Katherine S. Judge, Kaitlyn Lucas, Tayler Gowan, Patrick Stutz, Mu Shan, Laura Wilhelm, Tommy Parry, Shelley A. Johns

**Affiliations:** 1 Department of Medicine, School of Medicine, Indiana University, 1101 West 10th Street, Indianapolis, IN 46202 USA; 2 Division of General Internal Medicine, Geriatrics, and Palliative Care, Indianapolis, IN 46202 USA; 3 Regenstrief Institute, Indiana University Center for Aging Research, 1101 West 10th Street, Indianapolis, IN 46202 USA; 4 Department of Psychology, College of Sciences and Health Professions, Cleveland State University, 1836 Euclid Avenue, Cleveland, OH 44115 USA; 5 Regenstrief Institute, Center for Health Services Research, 1101 West 10th Street, Indianapolis, IN 46202 USA; 6 Department of Biostatistics, School of Medicine, Indiana University, 410 W. 10th Street, Suite 3000, Indianapolis, IN 46202 USA; 7 Department of Behavioral Medicine and Psychiatry, West Virginia University, 3200 MacCorkle Ave., SE, Charleston, WV 25304 USA; 8 Department of Psychology, Indiana University-Purdue University Indianapolis (IUPUI), 402 N Blackford St, Indianapolis, IN 46202 USA; 9 IUPUI Research in Palliative and End-of-Life Communication and Training Center, Indiana University-Purdue University Indianapolis, Indianapolis, USA

**Keywords:** Alzheimer’s disease, Anxiety; dementia, Caregiver, Acceptance and commitment therapy

## Abstract

**Background:**

Caregivers of patients with Alzheimer’s disease or a related dementia (ADRD) report high levels of distress, including symptoms of anxiety and depression, caregiving burden, and existential suffering; however, those with support and healthy coping strategies have less stress and burden. Acceptance and Commitment Therapy (ACT) aims to foster greater acceptance of internal events while promoting actions aligned with personal values to increase psychological flexibility in the face of challenges. The objective of this single-arm pilot, Telephone Acceptance and Commitment Therapy Intervention for Caregivers (TACTICs), was to evaluate the feasibility, acceptability, and preliminary effects of an ACT intervention on ADRD caregiver anxiety, depressive symptoms, burden, caregiver suffering, and psychological flexibility.

**Methods:**

ADRD caregivers ≥21 years of age with a Generalized Anxiety Disorder Scale (GAD-7) score ≥ 10 indicative of moderate or higher symptoms of anxiety were enrolled (*N* = 15). Participants received a telephone-based ACT intervention delivered by a non-licensed, bachelor’s-prepared trained interventionist over 6 weekly 1-h sessions that included engaging experiential exercises and metaphors designed to increase psychological flexibility. The following outcome measures were administered at baseline (T1), immediately post-intervention (T2), 3 months post-intervention (T3), and 6 months post-intervention (T4): anxiety symptoms (GAD-7; primary outcome); secondary outcomes of depressive symptoms (Patient Health Questionnaire–9), burden (Zarit Burden Interview), suffering (The Experience of Suffering measure), psychological flexibility/experiential avoidance (Acceptance and Action Questionnaire-II), and coping skills (Brief COPE).

**Results:**

All 15 participants completed the study and 93.3% rated their overall satisfaction with their TACTICs experience as “completely satisfied.” At T2, caregivers showed large reduction in anxiety symptoms (SRM 1.42, 95% CI [0.87, 1.97], *p* < 0.001) that were maintained at T3 and T4.

At T4, psychological suffering (SRM 0.99, 95% CI [0.41, 1.56], *p* = 0.0027) and caregiver burden (SRM 0.79, 95% CI [0.21, 1.37], *p* = 0.0113) also decreased.

**Conclusions:**

Despite a small sample size, the 6-session manualized TACTICs program was effective in reducing anxiety, suggesting that non-clinically trained staff may be able to provide an effective therapeutic intervention by phone to maximize intervention scalability and reach.

**Trial registration:**

Institutional Review Board (IRB) protocol #1904631305 version 05-14-2019. Recruitment began 06-14-2019 and was concluded on 12-09-2019.

Recruitment began 06-14-2019 and was concluded on 12-09-2019.

## Background

Of the more than 5 million people in the U.S. with Alzheimer’s disease or a related dementia (ADRD) [1], 75% are cared for by family caregivers [2]. Currently 16 million people in the U.S. are providing care to a family member or friend with ADRD [3, 4]. ADRD caregivers report higher distress, including symptoms of anxiety and depression [5–9], caregiving burden [10], and existential suffering [11], than caregivers of people with other chronic diseases, such as heart failure or cancer. Alarmingly, 60% of ADRD caregivers without an anxiety or depression diagnosis at the beginning of the caregiving journey develop one or both diagnoses within two years of caregiving [8]. This psychological morbidity is strongly associated with caregivers’ coping strategies [12, 13], such that support and healthy coping strategies are associated with less stress and burden [14–16].

Multi-component psychotherapeutic interventions, such as cognitive-behavioral therapy (CBT), have proven moderately effective in targeting caregiver distress and adaptive coping [17, 18]. Meta-analyses reveal that CBT reduces ADRD caregiver depressive symptoms; however, it is less effective for ADRD caregiver anxiety [17, 19, 20]. This may be due to misalignment between CBT goals and ADRD caregivers’ experiences. Specifically, CBT aims to challenge and modify dysfunctional thoughts viewed as antecedents of distress while increasing pleasant activities. For ADRD caregivers, the unchangeable nature of their situation and lack of control over their loved one’s cognitive decline or behaviors may make this impossible. Additionally, access to therapeutic interventions that are commonly delivered in-person is frequently cited as a limitation in ADRD caregiver interventions [17].

A different approach to supporting ADRD caregivers, acceptance-based coping, has been associated with reduced anxiety and depression for caregivers [21], suggesting that interventions focused on acceptance may provide a promising alternative for ADRD caregivers. A novel behavioral therapy, Acceptance and Commitment Therapy (ACT), offers therapeutic tools aimed at fostering greater acceptance of internal events (e.g., thoughts, feelings) while promoting actions aligned with personal values (e.g., being compassionate) to increase psychological flexibility in the face of challenges (e.g., care recipients’ progressive decline) [22, 23]. These tools may be useful to ADRD caregivers given the incurability of ADRD and the demands of caring for these individuals. ACT has proven beneficial for caregivers of children with autism [24] and life-threatening illnesses [25] and has been used to treat people with chronic pain [26] and anxiety [27]. Two European studies have investigated in-person ACT interventions for ADRD caregivers and found improved symptoms of anxiety and depression; however, perceived burden, suffering, and psychological flexibility were not measured [28, 29]. Additionally, research with US caregivers has yet to examine the feasibility and acceptability of implementing ACT with ADRD caregivers that is delivered via telephone.

The objective of this single-arm pilot was to evaluate the feasibility, acceptability, and preliminary effects of an ACT intervention on ADRD caregiver anxiety, depressive symptoms, burden, caregiver suffering, and psychological flexibility. For this study we define caregiver suffering as holistic construct that includes psychological distress, physical symptoms, and existential or spiritual suffering [11] and psychological flexibility as a measure of how caregivers relate to their thoughts and feelings. The Telephone Acceptance and Commitment Therapy Intervention for Caregivers (TACTICs) is a telephone- based behavioral intervention designed to increase caregivers’ capacity to connect with the present moment, accept difficult emotions, let go of unhelpful thoughts, take perspective, clarify values, and engage in meaningful action while navigating the challenges of caring for a family member with ADRD. To maximize the accessibility and scalability, clinical dissemination, and implementation potential of TACTICs, this study employed a non-licensed, bachelor’s-level interventionist to deliver the 6-week intervention via telephone.

## Methods

### Study design

The study was approved by the Indiana University Institutional Review Board (IRB#1904631305). Informed consent was obtained via phone from each participant upon enrollment and prior to any data collection.

The primary goal of this single arm pilot was to assess intervention feasibility and acceptability. We also assessed the impact of TACTICs on ADRD caregiver anxiety, depression, burden, suffering, psychological flexibility, and coping.

### Participants

TACTICs was designed for caregivers of older adults with ADRD; thus, persons with ADRD were not enrolled. Caregivers were included if they were ≥ 21 years of age, the primary caregiver for a family member with ADRD, able to provide informed consent, intended to continue caregiving for ≥12 months, able to communicate in English, willing to attend six weekly 1-h TACTICs sessions on the phone, and had a Generalized Anxiety Disorder Scale (GAD-7) score ≥ 10 indicating moderate or higher symptoms of anxiety [30, 31]. Caregivers were excluded if they self-reported that they were a non-family member, had a diagnosis of ADRD, or had a serious mental illness (e.g., bipolar or schizophrenia). Additionally, caregivers were not enrolled if their family member was living in long-term, supportive housing such as assisted living, personal care, or a nursing home. Caregivers were recruited from Indiana, USA from primary care clinics and the Aging Brain Care Program at Eskenazi Health; and primary care, geriatric psychiatry, and neurology clinics affiliated with Indiana University Health. Additionally, we recruited through community sites such as local organizations that sponsor support groups and other services for people with ADRD.

### Intervention and procedures

This pilot used a single arm design. Eligible caregivers were identified by active patient lists for participating clinics that were IRB approved recruitment sites. Caregivers (or emergency contacts or health care power of attorneys listed in patients’ electronic health records) were mailed an introductory letter about the TACTICs project. Potential participants who did not respond within 7–10 days after receiving the letter were contacted by a trained research assistant who presented more detailed information about the study and inquired about interest in participating. If the caregiver was interested, the research assistant administered an eligibility screener, which included the GAD-7. If caregivers met all eligibility criteria, they were again asked about interest. If they agreed to participate, the informed consent process, which included an assessment of decisional capacity with study-specific teach back questions to provide consent, was administered via telephone. Following the informed consent, the baseline assessment was completed, and all enrolled caregivers received paper copies of the Informed Consent form and a $20 gift card for completing the baseline assessment. The first TACTICs session was scheduled 1–3 weeks following the baseline assessment.

TACTICs is an ACT telephone-based intervention delivered to ADRD caregivers by a non-licensed, bachelor’s-prepared trained interventionist. As an ACT intervention, TACTICs is a mindfulness-based behavioral therapy that incudes the main processes of ACT; developing acceptance of unwanted private experiences which are out of persons control and recognizing a person’s commitment and action toward living a valued life.

ACT interventions have been shown to be effective with a diverse range of clinical conditions [32]. The goal of the program is to enhance psychological flexibility through practice of six core skills—acceptance, cognitive defusion, mindful awareness of the present moment, self-as-context (perspective taking), values clarification, and committed action. Psychological flexibility focuses on connecting with the present moment rather than avoiding unwanted internal experiences and engaging in behavior aligned with one’s values [33, 34]. Notably, psychological flexibility is theoretically linked to improvements in anxiety [27], depressive symptoms [35], and wellbeing [35]. Prior to the first session, caregivers received a TACTICs binder for use during sessions that included reading materials, worksheets, and handouts summarizing session topics. The 6-week intervention consisted of 1-h telephone sessions that included engaging experiential exercises and metaphors designed to increase psychological flexibility through practice of one or more of the six skills in each session (see Table 1 for session descriptions). The interventionist guided caregivers in brief mindfulness meditation practices that encouraged non-judgmental awareness of thoughts, feelings, and bodily sensations in the present moment in each session. To strengthen psychological flexibility, participants were invited to practice mindfulness at home in between sessions using 10-min audio recordings available via a computer download or compact disc, according to each participant’s preference. Caregivers also identified deeply-held values to serve as a guide when choosing how to spend limited time or energy and set values-based action goals each week.

**Table 1:** TACTICs Intervention Sessions

Session	Theme, Mindfulness Practice, and Home Practice
**1**	**Fostering Contact with the** ***Present-Moment*:** Cultivate present-moment awareness; explore caregiving stressors and usual responses; see opportunities for wise action **Mindfulness**: Body Scan **Home practice**: Body Scan daily; eat one meal mindfully; mindfulness of one daily activity
**2**	***Values*** **and Meaningful Connections**: Value-based living; mindful acceptance to promote values-consistent behavior **Mindfulness**: Abbreviated Body Scan with Awareness of Breath; Watching the Sky **Home practice**: Choice of daily mindfulness practice; values-based action worksheet
**3**	**Being Here for the Life You Have: Knowing** ***Self as Context***: Observe inner experiences without getting “hooked” to lessen suffering and live with purpose **Mindfulness**: Body Scan; Leaves on a Stream **Home practice**: Body Scan daily; Passengers on a Bus worksheet; Caregiver thinking diary
**4**	**Making Wise Choices:** ***Acceptance*** **&** ***Defusion***: Differentiate *having* a thought from *buying* a thought; acceptance/willingness differs from control/avoidance **Mindfulness**: Awareness of Breath; 3-Step Compassion practice **Home practice**: Alternate Body Scan and 3-Step Compassion practice daily
**5**	**Embracing the** ***Present Moment*** **and Choosing** ***Values*-*Based Action*** **on the Path to Vital Living**: Notice how body and mind feel at pleasant and unpleasant times; use values as a guide for meaningful living **Mindfulness**: Body Scan with “It’s like this…yes”; Welcome Anxiety My Old Friend **Home practice**: Choice of daily mindfulness practice; Values Form; Embracing the Unwanted
**6**	***Committed Action*** **and Existential Well-Being**: Differences between pre-study anxiety coping vs. newer options; reinforce action plans **Mindfulness**: Minimally-guided Sitting Meditation; Lovingkindness Meditation **Home practice**: Reinforce possibilities to support continued practice of skills; resource flier

The bachelor’s-level interventionist (TMG) has a four-year degree in Psychology but is not a licensed therapist or psychologist. She was trained by a doctoral level clinical health psychologist (SAJ) using didactics, readings, live demonstrations, and role-plays. The interventionist also received supervision throughout the study from a master’s level clinician (TDP) with ACT training. A total of 23 (25.6%) of the audio-recorded TACTICs sessions were assessed for fidelity to the intervention manual using a structured fidelity checklist similar to the one used in our previous ACT interventions [36]. The average fidelity rating across all sessions rated was 98.6% (SD = 0.03), suggesting the interventionist delivered TACTICs in a manner that was highly adherent to the intervention manual.

### Data collection and measures

All data were collected from caregivers via phone by a trained research assistant and entered online into a secure REDCap (Research Electronic Data Capture) database.

To assess preliminary efficacy of the intervention, psychometrically validated outcome measures were administered at baseline (T1), immediately post-intervention (T2), 3 months post-intervention (T3), and 6 months post-intervention (T4). At baseline (T1), social and demographic data were also collected, including age, sex, race, ethnicity, relationship to the ADRD patient, frequency of contact with the patient, geographic distance from the patient, caregiver education level, and annual income. Severity of cognitive impairment for each participant’s care recipient was also assessed at T1 using the Dementia Severity Rating Scale (DSRS) [37]. Feasibility was measured by calculating the enrollment, completion of intervention, attrition, and completion rates of outcome assessments through T4. Acceptability was measured by caregiver responses to a 7-item TACTICs survey that assessed satisfaction with TACTICs at T2.

The Generalized Anxiety Disorder Scale (GAD-7) [30, 31] consists of 7 items that assessed anxiety symptoms (primary outcome) at each time point [30, 31, 38]. Depressive symptoms were measured with the Patient Health Questionnaire–9 (PHQ-9) that consists of 9 items that assess somatic and non-somatic symptoms of depression [39, 40]. Caregiver burden was measured with the Zarit Burden Interview (ZBI), which includes 22 items that measure the objective and subjective burden experienced by family caregivers [38, 41, 42]. Caregiver suffering was assessed with The Experience of Suffering measure that contains 33 items across three subscales: physical (9 items), psychological (15 items), and existential (9 items) suffering [11]. Psychological flexibility and its opposite, experiential avoidance, were measured at each time point with the 7-item Acceptance and Action Questionnaire-II (AAQ-II) [43]. Coping skills and styles of caregivers were measured with the Brief COPE, a 28-item measure of 14 coping strategies used in response to stressors [44]. All measures have been statistically validated and have demonstrated good internal consistency in prior trials [31, 42].

### Data analysis

Descriptive statistics for caregivers’ social and demographic characteristics were summarized as frequency and percent for categorical variables, as mean and standard deviation for normal continuous variables, including their relationship to the person with ADRD and the severity of the care recipients’ ADRD, were calculated. TACTICs feasibility measures included at least 50% of eligible caregivers enrolling in the study and attendance rates of 70% or greater across the six TACTICs sessions. Acceptability was assessed to be that at least 70% of caregivers enrolled in the study completed the study through T4 and at least 70% of enrolled caregivers reported being mostly to completely satisfied with their experience in TACTICs [45].

The standardized response mean (SRM) effect size for the outcomes was calculated to assess the magnitude of intervention effects at T2, T3, and T4. To determine SRM, mean change in T2, T3, and T4 scores relative to baseline (T1) was calculated and divided by the standard deviation (SD) of change. The 95% confidence intervals (CIs) were computed for each caregiver (caregiver’s mean change divided by the sample’s SD of change scores). The SAS MEANS procedure with the LCLM and UCLM options were used to compute the lower and upper 95% confidence limits for the SRM statistic. The primary efficacy-related goal of this pilot was to estimate effect sizes, and the 2-sided paired *t* test was used to determine significant (*P* < 0.05) responsiveness over time. Due to the small sample, marginal significance (0.05 < *P* < 0.10) is also reported. Standardized response means of 0.2, 0.5, and 0.8 indicated small, medium, and large effect sizes, respectively [46]. Analyses were performed using SAS version 9.4 (SAS Institute, Inc., Cary, NC).

## Results

### Social and demographic characteristics

Social and demographic characteristics of the 15 caregivers who completed TACTICs are shown in Table 2. On average, caregivers were 68 years old, and most (73%) were caregiving for their spouse with ADRD; 80% were female, and all reported their race as non-Hispanic and white. The majority (93%) were caring for an individual with mild to moderate ADRD and reported their own health status as good (53%), very good (40%) or excellent (6.7%). At baseline, 87% had mild or moderate depressive symptoms and all (100%) had high levels of caregiver burden as measured by the Zarit Burden Index. Given that clinically significant anxiety was required for eligibility, 73% had moderate anxiety and 27% had severe anxiety at baseline. Two caregivers had their family member move into long-term care during their participation in TACTICs. Given that this was a pilot and that family caregivers continue to provide care and support when their family member moves into long-term care, the remained in the study [47].

**Table 2:** Caregiver Social and Demographic Characteristics

Variable	Caregivers ***n*** = 15
**Completed all 6 TACTICs sessions,** *n* (%)	15 (100)
**Age in years**, mean (SD)	68.85 (11.70)
**Sex**, *n* (%)	
Female	12 (80)
**Ethnicity and race**, *n* (%)	
Non-Hispanic White	15 (100)
**Education**, *n* (%)	
Not a college graduate	6 (40)
College graduate	9 (60)
**Self-reported income situation**, *n* (%)	
Do not have enough to make ends meet	1 (6.7)
Have just enough to make ends meet	4 (26.7)
Comfortable	10 (66.6)
**Recruitment location**, *n* (%)	
Clinical sites	3 (20)
Community sites	12 (80)
**Caregiver relationship to ADRD patient**, *n* (%)	
Spouse	11 (73.3)
Adult child or child-in-law	3 (20)
Sibling	1 (6.67)
**Severity of ADRD of caregiver’s care recipient**, *n* (%)	
Mild	4 (26.7)
Moderate	10 (66.6)
Severe	1 (6.7)
**Participate in caregiver support group during TACTICs**	
Yes	4 (26.7)
**Participate in individual therapy or counseling during TACTICs**	
Yes	2 (13.3)
**Caregiver self-reported health status**, *n* (%)	
Excellent	1 (6.7)
Very good	6 (40)
Good	8 (53.3)

### Feasibility and acceptability

**Fig. 1:**
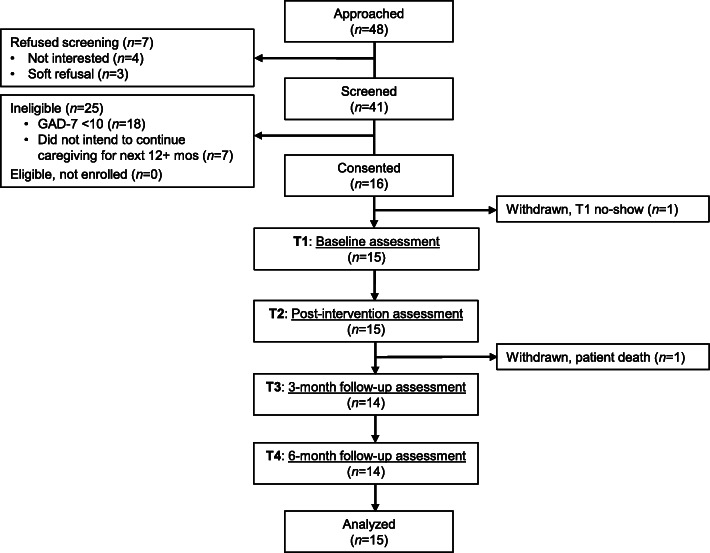
Consolidated Standards of Reporting Trials (CONSORT) flowchart, including number of participants assessed at each time point

Over 25 weeks, 48 caregivers were approached. Seven (14.5%) caregivers refused to be screened for eligibility while 41 (85.4%) agreed (see Fig. 1). Of the 41 caregivers screened for eligibility, 16 (39%) were eligible and all 16 (100%) enrolled in the study. Twenty-five (61%) caregivers were ineligible, with GAD-7 scores < 10 representing the primary reason for ineligibility.

Retention across the study timeframe was high. One caregiver withdrew between consent and T1 (prior to beginning TACTICs) and one caregiver withdrew between T2 and T3, resulting in an overall retention rate of 87.5% at T4. With respect to adherence to the TACTICs protocol, 100% of caregivers who began TACTICs (*n* = 15) completed all six sessions.

To determine program acceptability, participants rated six questions about their satisfaction with TACTICs. Using a 5-point Likert scale with “1” being “extremely unsatisfied” and “5” being “extremely satisfied,” participants were asked to rate their satisfaction with TACTICs. One hundred percent of participants rated their overall satisfaction with their TACTICs experience as a 9 or 10 on a 10-point scale (Table 3).

**Table 3:** Caregiver Satisfaction with TACTICs ^a^

Survey Item		Caregivers (***n*** = 15)	
		Mean	SD
Overall, how satisfied are you with your experience in the TACTICs program?	1 = Not at all satisfied 10 = Completely Satisfied	9.14	0.95
How satisfied are you with the number of sessions?	1 = Extremely unsatisfied 5 = Extremely Satisfied	4.50	0.85
How satisfied are you with the length of the sessions?		4.43	0.76
How satisfied are you with the topics of the sessions?		4.79	0.43
How satisfied are you with the skill of the study therapist?		4.79	0.43
How satisfied are you with the reading materials and worksheets you received?		4.50	0.65
How satisfied are you with the mindfulness recordings you received?		4.57	0.65

^a^ All items were asked at T2

### Intervention effects

Table 4 shows preliminary intervention effects for caregivers. At T2, caregivers showed a significantly large reduction in anxiety symptoms (SRM 1.42, 95% CI [0.87, 1.97], *p* < 0.001) and a medium reduction in caregiver physical suffering that approached statistical significance (SRM 0.50, 95% CI [0.05, 1.05], *p* = 0.07).

**Table 4:** Caregiver Outcomes

Outcomes	T1 Mean (SD) ***n*** = 15	T2 Mean (SD) ***n*** = 15	T3 Mean (SD) ***n*** = 14	T4 Mean (SD) ***n*** = 14	T1 – T2 SRM 95% CI	***P***-value	T1-T3 SRM 95% CI	***P***-value	T1-T4 SRM 95% CI	***P***-value
**Distress**										
Anxiety	13.33 (2.79)	8.00 (3.21)	7.00 (3.78)	6.07 (3.36)	1.42 (0.87, 1.97)	< 0.0001	1.28 (0.71, 1.86)	0.0003	1.94 (1.36, 2.51)	< 0.0001
Depressive symptoms	7.47 (3.20)	5.80 (3.12)	6.07 (4.03)	5.57 (3.74)	0.42 (−0.14, 0.97)	0.1299	0.34 (− 0.24, 0.92)	0.2277	0.44 (− 0.14, 1.01)	0.1271
Caregiver Burden	41.73 (11.63)	39.13 (15.53)	39.00 (12.44)	34.50 (15.58)	0.31 (− 0.24, 0.86)	0.2486	0.40 (− 0.17, 0.98)	0.1542	0.79 (0.21, 1.37)	0.0113
Psychological Flexibility	18.53 (7.59)	17.27 (8.04)	18.29 (8.47)	16.29 (8.25)	0.24 (− 0.31, 0.79)	0.3677	0.10 (− 0.48, 0.68)	0.7080	0.38 (− 0.20, 0.96)	0.1800
**Caregiver suffering**										
Physical suffering	7.13 (2.26)	6.13 (3.11)	6.64 (3.91)	6.14 (3.46)	0.50 (− 0.05, 1.05)	0.0733	0.11 (− 0.47, 0.68)	0.6977	0.27 (− 0.31, 0.84)	0.3390
Psychological suffering	14.07 (4.98)	13.67 (6.03)	13.29 (5.55)	10.50 (4.11)	0.11 (− 0.44, 0.66)	0.6786	0.13 (− 0.45, 0.71)	0.6396	0.99 (0.41, 1.56)	0.0027
Existential suffering	15.00 (3.78)	15.20 (3.55)	15.93 (3.71)	16.07 (3.89)	−0.07 (− 0.63, 0.48)	0.7828	− 0.48 (−1.06, 0.10)	0.0966	−0.47 (−1.05, 0.11)	0.1007
**Coping**										
Self-distraction	5.93 (1.22)	5.53 (1.36)	5.21 (1.19)	6.07 (1.54)	0.38 (−0.17, 0.93)	0.1643	0.54 (− 0.04, 1.12)	0.0650	− 0.08 (− 0.66, 0.50)	0.7701
Denial	2.00 (0)	2.13 (0.35)	2.21 (0.58)	2.71 (1.27)	−0.38 (− 0.93, 0.17)	0.6164	−0.37 (− 0.95, 0.21)	0.1894	−0.56 (− 1.14, 0.01)	0.0548
Behavioral disengagement	2.47 (0.83)	2.13 (0.35)	2.36 (0.63)	2.50 (1.02)	0.41 (−0.15, 0.96)	0.1362	0.10 (− 0.48, 0.68)	0.7207	− 0.07 (− 0.64, 0.51)	0.8069
Acceptance coping	6.60 (1.18)	6.53 (1.81)	7.00 (1.18)	6.50 (1.56)	0.05 (− 0.50, 0.60)	0.8494	−0.38 (− 0.96, 0.19)	0.1739	0.08 (− 0.50, 0.66)	0.7646
Active coping	6.13 (1.46)	6.00 (1.36)	6.43 (1.74)	6.21 (1.42)	0.10 (− 0.46, 0.65)	0.7090	−0.27 (− 0.84, 0.31)	0.3356	−0.15 (− 0.73, 0.43)	0.5830

*Abbreviations*: *CI* confidence interval, *SD* standard deviation, *SRM* standardized response mean, *T1* baseline, *T2* immediately post-intervention, *T3* 3 months post-intervention, *T4* 6 months post-intervention

At T3 and T4, effects were strengthened for anxiety symptoms. Caregivers showed large, statistically significant improvements in GAD-7 scores at T3 (SRM 1.28, 95% CI [0.71, 1.86], *p* = 0.0003) and T4 (SRM 1.94, 95% CI [1.36, 2.51], *p* < 0.0001). At T4, statistically significant decreases in caregiver psychological suffering (SRM 0.99, 95% CI [0.41, 1.56], *p* = 0.0027) and caregiver burden (SRM 0.79, 95% CI [0.21, 1.37], *p* = 0.0113) were observed.

Although they did not reach statistical significance, the trends for the outcomes of physical suffering and the coping subscales of self-distraction and denial are important to consider when thinking about future research studies with larger samples. Specifically, physical suffering showed a decrease between T1 and T2 (SRM 0.50, 95% CI (0.05, 1.05) *p* = .073); self-distraction showed a decline between T1 and T3 (SRM 0.54, 95% CI (− 0.04, 1.12) *p* = .06); and denial showed an improvement between T1 and T4 (SRM -0.56, 95% CI (− 1.14, 0.01) *p* = .054).

## Discussion

This pilot of TACTICs, an ACT-derived intervention for ADRD caregivers with clinically significant anxiety, has several important findings. First, a 6-week ACT intervention delivered remotely, via telephone, is feasible and highly acceptable among ADRD caregivers. Second, an ACT intervention for this population can be successfully tailored to individual caregivers’ experience and be delivered by bachelor’s-level, non-licensed personnel with high fidelity. Third, preliminary effects suggest that TACTICs may significantly reduce moderate-to-severe anxiety, a common and disruptive symptom among ADRD caregivers [31]. Lastly, these large, statistically significant, and clinically meaningful reductions in anxiety symptoms were demonstrated at each follow-up and sustained at 6 months post-intervention [48, 49]. Despite the small sample size and limited power, efficacy tests generally showed statistically significant results for anxiety and marginally significant effect sizes for caregiver burden.

Possibly the most important finding from this pilot was the willingness of ADRD caregivers to participate in TACTICs with high adherence and satisfaction. Among the 15 caregivers who enrolled and completed the baseline assessment, 100% competed all six sessions, demonstrating an extremely high level of protocol adherence and acceptability. This includes two caregivers who experienced their family member with ADRD moving into long-term care during their time in the TACTICs project. Additionally, 100% of caregivers rated satisfaction with the TACTICs program as ≥9 on a 1 to 10-point scale, and 100% endorsed that the TACTICs program “quite a bit” or “very much” helped them cope more effectively with caring for their family member with ADRD. These results speak to the validation of TACTICs for each of these caregivers and their unique experiences. The majority (92.9%) stated they were “very much” confident in recommending TACTICs to other ADRD caregivers. Collectively, these results suggest that TACTICs, delivered remotely by a bachelor’s-level non-licensed interventionist, was well-received by participants. These findings are important in establishing TACTICs as a scalable protocol that is both clinically relevant and has high implementation potential. Notably, 73% of participating caregivers were spouses of the ADRD patient, suggesting our sample consisted mainly of older adults. Unbound by geographical constraints, TACTICs has greater potential to reach older and rural participants who may lack access to well-networked urban care facilities (e.g., Indianapolis-based care team can deliver TACTICs to anyone in the U.S.). These findings are also important regarding alternative forms of delivering TACTICs, such as on-line and synchronous with an interventionist or possibly asynchronous with curated modules that caregivers can access whenever they desire.

Although the results of this pilot are promising, some limitations are important to note for interpreting these results and planning for a future, larger trial of an ACT intervention for ADRD caregivers. First, TACTICs was tested outside traditional care or support systems that are currently in place for ADRD caregivers [50]. For example, caregivers were recruited from a variety of settings, and each had their unique set of access to services that could support or not support their caregiving. We found that almost 30% of caregivers also participated in support groups and 13% were receiving individual counseling or therapy during the intervention, highlighting that TACTICs was deemed helpful to caregivers with and without other supportive services [51–54]. It is unclear whether other outstanding needs of caregivers made TACTICs more or less effective in impacting their anxiety or burden. Second, the majority of caregivers (66.6%) in the study were providing care for individuals with moderate ADRD. Future studies should include equal representation of caregivers across the severity of the illness to examine the impact of TACTICs along with potential interaction effects based on the level of cognitive impairment.

With respect to the larger caregiving literature, TACTICs provides a novel avenue for addressing key issues faced by caregivers of individuals with ADRD. Specifically, TACTICs equips caregivers with specific skills (e.g., acceptance, defusion) for managing unchangeable personal experiences. For many caregivers, caring for a loved one with ADRD is overwhelming and requires a specific set of psychological skills for managing their own internal experiences. TACTICs was designed to empower caregivers to cope more adaptively with their stressful realities by increasing overall psychological flexibility. Studies have found psychological flexibility to be a significant buffer against psychological distress (i.e., anxiety, depressive symptoms) in family caregivers [55]. TACTICs had a small effect on psychological flexibility (SRMs = 0.10–0.38 across all follow-up assessments). Notably, our sample expressed relatively high levels of psychological flexibility at baseline, which may have limited the magnitude of effect on this outcome. The small effect may also be attributable to our use of a general measure of psychological flexibility (AAQ-II) rather than one that was adapted for caregivers. Conceptually, as an intervention, TACTICs may be addressing elements of the caregiving experience that have yet to be truly understood and intervened upon for caregivers of individuals with ADRD. For example, a caregiver may benefit from learning how to improve communication with their loved one and manage difficult behaviors. Despite these benefits, however, caregivers may still experience distressing thoughts and feelings that they attempt to suppress or avoid. Although these psychologically inflexible strategies for coping with painful internal experiences are common, they can result in symptoms of anxiety, depression, burden, and other negative psychosocial outcomes. TACTICs offers a promising approach for addressing these issues that are not traditionally included in current caregiver interventions.

## Conclusions

ADRD caregivers experience high rates of anxiety, and they are willing to participate in telephone-delivered interventions that include mindfulness and values-based action to target their anxiety. Despite a small sample size, the 6-session manualized TACTICs program was effective in reducing anxiety, suggesting that non-clinically trained staff may be able to provide an effective therapeutic intervention to maximize intervention scalability and reach.

## Data Availability

The protocol and datasets used and analyzed during the current study are available from the corresponding author on reasonable request.

## Abbreviations

AAQ-II: Acceptance and Action Questionnaire
ACT: Acceptance and Commitment Therapy
ADRD: Alzheimer’s Disease and Related Dementias
CI: Confidence interval
GAD-7: General Anxiety Disorder Scale
IRB: Institutional Review Board
PHQ-9: Patient Health Questionnaire
ZBI: Zarit Burden Interview
